# Anti-Inflammatory Activity of Heterocarpin from the Salt Marsh Plant *Corydalis heterocarpa* in LPS-Induced RAW 264.7 Macrophage Cells

**DOI:** 10.3390/molecules200814474

**Published:** 2015-08-10

**Authors:** You Ah Kim, Chang-Suk Kong, Hyo Hyun Park, Eunkyung Lee, Mi-Soon Jang, Ki-Ho Nam, Youngwan Seo

**Affiliations:** 1 Division of Marine Bioscience, Korea Maritime & Ocean University, Busan 606-791, Korea; E-Mail: ahyou2@hanmail.net; 2 Department of Food and Nutrition, Silla University, Busan 617-736, Korea; E-Mail: cskong@silla.ac.kr; 3 Research and Development Division, Korea Promotion Institute for Traditional Medicine Industry, Gyeongsan 712-260, Korea; E-Mails: qkrgygus@empal.com (H.H.P.); eklee@ynu.ac.kr (E.L.); 4 Food and Safety Research Center, National Fisheries Research & Development Institute, Busan 619-705, Korea; E-Mails: suni1@korea.kr (M.-S.J.); dennis011@korea.kr (K.-H.N.); 5 Ocean Science & Technology School, Korea Maritime & Ocean University, Busan 606-791, Korea

**Keywords:** *Corydalis heterocarpa*, heterocarpin, furochromone, anti-inflammatory effect

## Abstract

The inhibitory effect of three chromones **1**–**3** and two coumarins **4**–**5** on the production of nitric oxide (NO) was evaluated in LPS-induced RAW 264.7 macrophage cells. Among the compounds tested heterocarpin (**1**), a furochromone, significantly inhibited its production in a dose-dependent manner. In addition, heterocarpin suppressed prostaglandin E_2_ (PGE_2_) production and expression of cytokines such as inducible nitric oxide synthase (iNOS), cyclooxygenase-2 (COX-2), tumor necrosis factor-α (TNF-α), interleukin-1β (IL-1β) and interleukin-6 (IL-6).

## 1. Introduction

Halophytes are naturally salt-tolerant plants that may be potentially useful as new sources of biologically active substances. These plants are exposed in their habitats to various abiotic constraints (salinity, drought, heat/cold, luminosity and other harsh environmental conditions), and as a result, they have had to adapt to their environment by developing specific responses which often include the synthesis of unusual secondary metabolites. *Corydalis heterocarpa* is a salt-tolerant plant species that grows on sandy seashores throughout the western coastal area of South Korea. The genus *Corydalis* is composed of more than 400 species in Eurasia and North America. For centuries some *Corydalis* species have been long used for the treatment of ailments in Asian countries including China, Korea, and Japan. *C.* *heterocarpa* has been also used in Korean folk medicine to treat labor pains, spasms, boils, and dysentery. Nevertheless, only a few studies of this species have been reported. To date *C.* *heterocarpa* has been shown to possess UVB-protection, anti-tumor, anti-oxidant and anti-inflammatory effects. These effects seem to be due to coumarins, promoting them as a source of biofunctional products [1,2,3,4,5]. Like other salt marsh plants, a high content of phytochemicals which are crucially needed to endure the highly salinic environmental conditions is a characteristic of *C. heterocarpa*. Coupled with the high potential of phytochemicals and high rates of nutritional consumption, the utilization of *C. heterocarpa* as a functional food has been suggested. In this regard, research on the secondary metabolites and bioactivities of the *C. heterocarpa* has not been done intensely enough.

In the present study, the anti-inflammatory effect of three chromones **1**–**3** and two coumarins **4**–**5** isolated from *C. heterocarpa* was assessed by measuring NO production in LPS-stimulated RAW 264.7 cells. Furthermore, production of iNOS, PGE_2_, TNF-α, IL-1β, IL-6, and COX-2 on heterocarpin-treated RAW 264.7 macrophage cells was investigated.

## 2. Results and Discussion

### 2.1. Isolation and Structure Determination of Compounds ***1**–**5***

**Figure 1:**
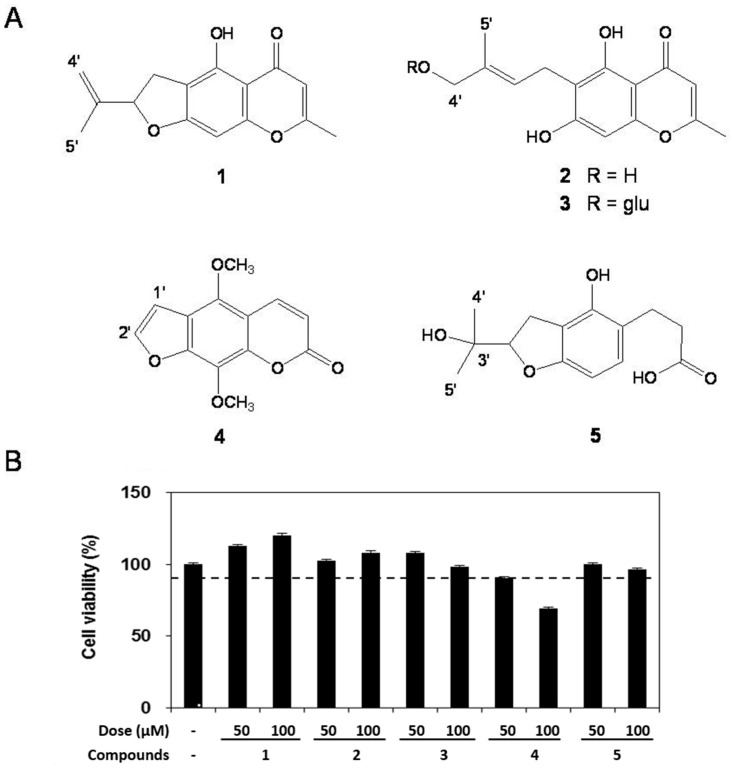
Chemical structure (**A**) and effect on cell viability (**B**) of compounds **1**–**5** isolated from *Corydalis heterocarpa* on RAW 264.7 cells. RAW 264.7 cells were treated with 50 and 100 μM of compounds **1**–**5** for 1 h prior to the addition of LPS (100 ng/mL), and the cells were further incubated for 24 h. Data are presented as means ± SD of three independent experiments.

Shade-dried whole plants of *Corydalis heterocarpa* were extracted twice overnight with CH_2_Cl_2_ and MeOH at room temperature. The combined crude extracts were concentrated *in vacuo* at 40 °C to leave a dark brown gum and then partitioned between CH_2_Cl_2_ and H_2_O. Each layer was further partitioned with *n-*hexane/85% aq. MeOH and *n-*BuOH/H_2_O, respectively. The *n-*BuOH and 85% aq. MeOH fractions were purified by various column chromatographies, TLC and HPLC methods using different solvent combinations to yield three chromones and two coumarins (Figure 1A).

Compounds **1**–**5** were identified by a combination of spectroscopic analysis and comparison with previously reported data as heterocarpin (**1**), cnidimol A (**2**), cnidimoside A (**3**), isopimpinellin (**4**) and hyunganol II (**5**), respectively [6,7,8,9].

### 2.2. Cell Viability

We first measured the cytotoxicity of compounds **1**–**5** in RAW 264.7 cells by using the MTT assay. The result showed compounds **1**–**5** at the concentrations used (50 and 100 μM) had no cytotoxic effect, except compound **4** at a concentration of 100 μM (Figure 1B).

### 2.3. Effect of Compounds ***1**–**5*** on LPS-induced NO Production and iNOS Expression

**Figure 2:**
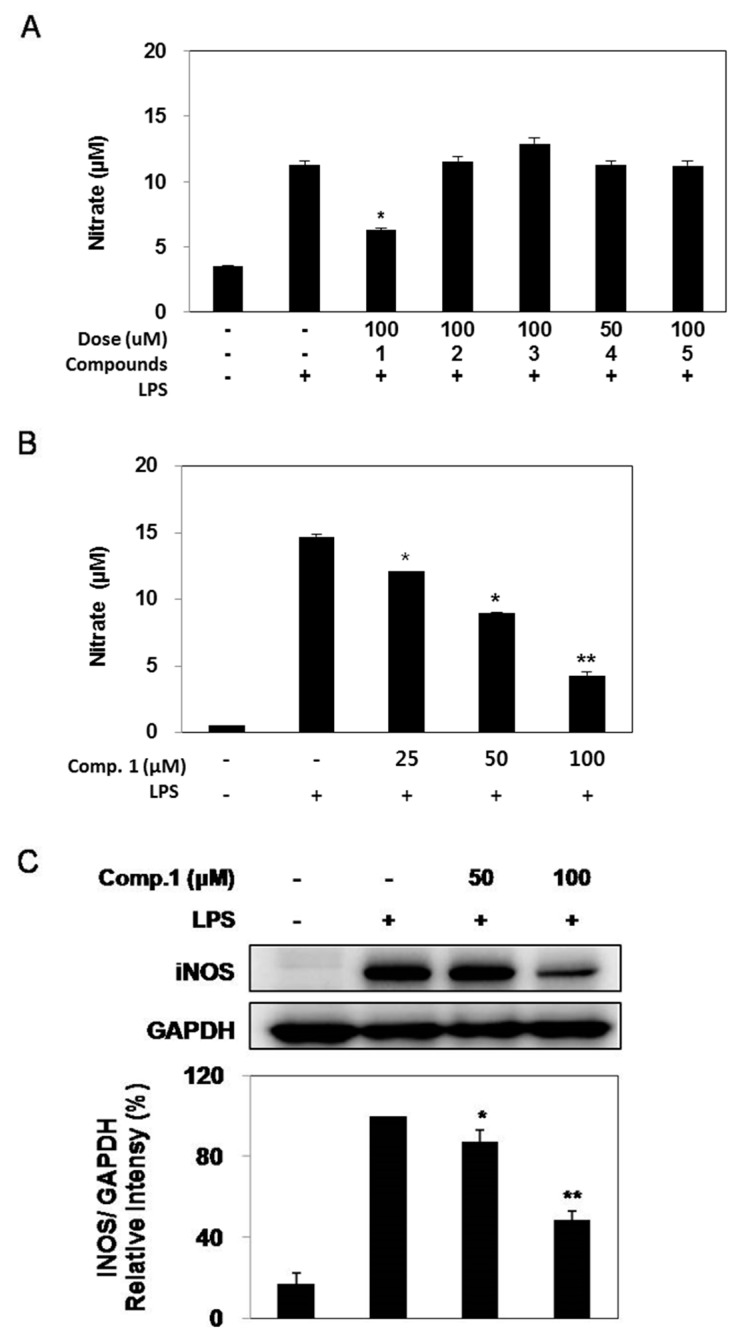
Effects of compounds **1**–**3**, **5** (100 μM) and **4** (50 μM) (**A**), and compound **1** (25–100 μM) (**B**) on NO production by means of nitrite concentration and iNOS expression by means of protein expression levels (**C**) in LPS induced RAW 264.7 cells. RAW 264.7 cells were cultured with LPS (100 ng/mL) in the presence or absence of compounds **1**–**5** for 24 h to determine the level of NO. The data represent the mean ± SD of three separate experiments (significant as compared to control. * *p* < 0.05 and ** *p* < 0.01).

To investigate the inhibitory effect of compounds **1**–**5** on NO production, RAW 264.7 cells were pretreated with each compound for 1 h prior to stimulation with LPS. Following 24 h of LPS stimulation, the levels of NO production in the culture media were measured. As shown in Figure 2A, LPS stimulation resulted in a marked induction of NO production when compared to the untreated cells and pretreatment with compound **1** suppressed the NO production with an IC_50_ value of 66.6 μM (Figure 2B), whereas other compounds did not reduce nitrite levels (Figure 2A). To determine whether suppression of NO production by compound **1** was due to reduced iNOS protein expression, cells were pretreated with different concentrations of compound **1** and stimulated with LPS for 24 h. The significant induction of iNOS protein by LPS stimulation was inhibited by compound **1** in a dose-dependent manner (Figure 2C). These results showed that compound **1** inhibited NO production by the decreased iNOS protein expression.

### 2.4. Effect of Compound ***1*** on LPS-Induced PGE2 Production and COX-2 Expression

**Figure 3:**
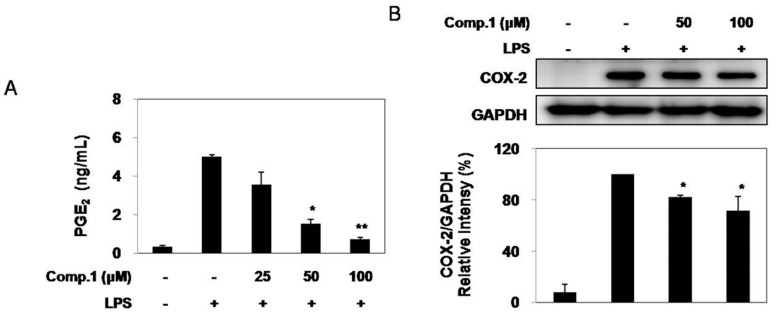
Effects of compound **1** on production of PGE_2_ (**A**) and COX-2 expression (**B**) in LPS induced RAW 264.7 cells. RAW 264.7 cells were treated with different concentrations (25, 50, 100 μM) of compound **1** for 1 h prior to stimulation with 100 ng/mL of LPS for 24 h. Control values were obtained in the absence of LPS or compound **1**. The values are the mean ± SD of three independent experiments (significant as compared to control. * *p* < 0.05 and ** *p* < 0.01).

To further elucidate the anti-inflammatory response of compound **1**, we examined the inhibitory effect of compounds **1** on PGE_2_ production following LPS stimulation in RAW 264.7 cells. Briefly, the cells were pretreated with various concentrations of compound **1** for 1 h and then stimulated with LPS. After incubation for 24 h, the production of PGE_2_ from the culture supernatant was measured using ELISA. As shown in Figure 3A, the amount of PGE_2_ in the culture supernatant increased with LPS stimulation, and then this increase was reduced by treatment with compound **1** in a dose-dependent manner (*p* < 0.05). To investigate whether the inhibitory effects of compound **1** on PGE_2_ production are related to the modulation of the COX-2 expression, we evaluated the COX-2 protein levels using Western blot analysis. The COX-2 protein was not detected in the absence of LPS stimulation, but the levels of COX-2 were induced after LPS exposure. As shown in Figure 3B, compound **1** reduced LPS-stimulated COX-2 protein expression.

### 2.5. Effects of Compound 1 on Pro-Inflammatory Cytokine Production

**Figure 4:**
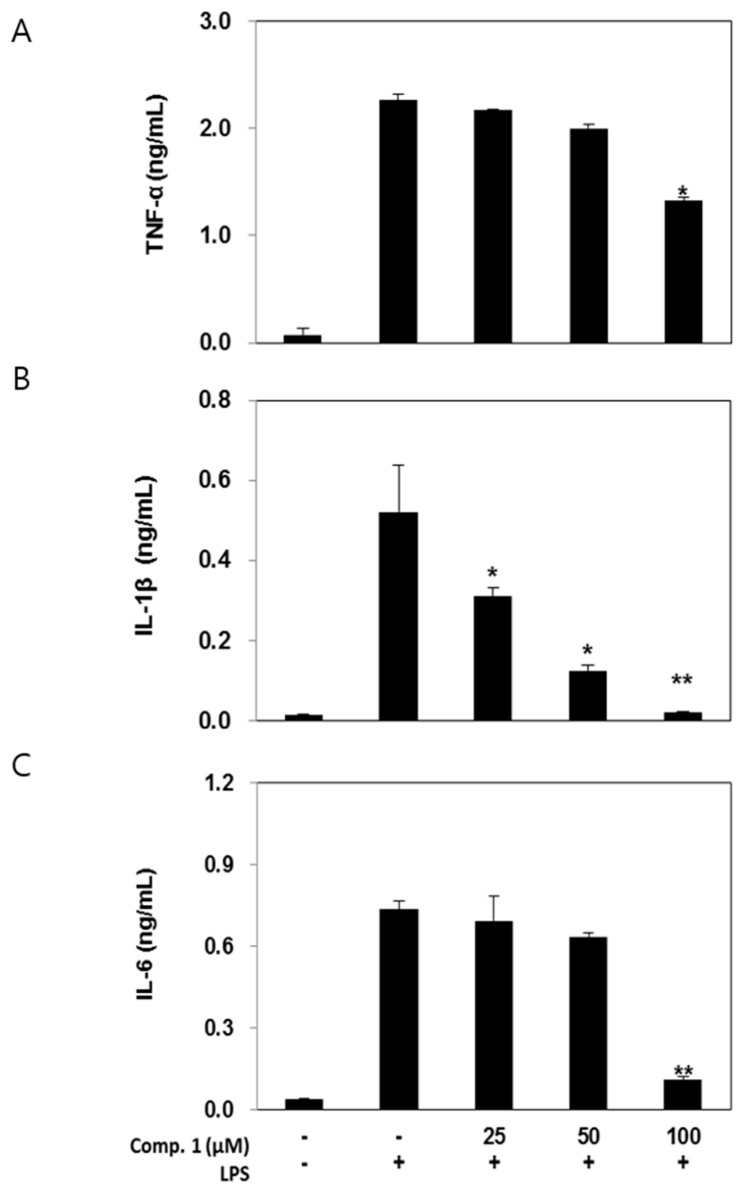
Effect of compound **1** on the production of cytokines stimulated by LPS. Production of TNF-α (**A**), IL-1β (**B**) and IL-6 (**C**) were measured in the medium of RAW 264.7 cells cultured with LPS (100 ng/mL) in the presence or absence of compound **1** for 24 h. Data represent the mean ± SD with three separate experiments (significant as compared to control. * *p* < 0.05, ** *p* < 0.01).

To further determine the anti-inflammatory effect of compound **1**, the secreted pro-inflammatory cytokines, such as TNF-α, IL-1β and IL-6 were measured by ELISA. Treatment of RAW 264.7 cells with LPS alone resulted in a marked induction of cytokine production when compared to the untreated cells (*p* < 0.001) (Figure 4). However, the pretreatment of compound **1** at 100 μM significantly decreased TNF-α levels compared to the supernatant of the LPS-stimulated cells (*p* < 0.05). In addition, the levels of IL-1β and IL-6 were increased in the culture supernatant of the LPS-stimulated cells whereas pretreatment with compound **1** at 50 and 100 μM resulted in a decrease in cytokine production (*p* < 0.05 or 0.01).

### 2.6. Discussion

In recent years, there has been much interest in and research on the influence of natural products on several diseases, including aging-related illness, cancer, neurodegenerative disease, and cardiovascular disease. *Corydalis* species have been extensively investigated for this purpose, and have yielded alkaloids, isoquinoline alkaloids, and cyanidin glycosides as their secondary metabolites [10,11,12]. *Corydalis heterocarpa* has been widely used as a folk medicine for treatment of labor pains, spasms, boils and dysentery [2,3,4,5]. Macrophages play an important role in inflammatory disorders through the release of factors such as NO, cytokines, and prostaglandin mediators in the immune system [13,14,15,16]. The inducible form of NO is generated from L-arginine by iNOS. NO, a free radical, plays important roles in a variety of physiological functions such as host defense, vasodilation, and regulation of cellular functions [17,18]. However, overproduction of NO by iNOS further enhances cyclooxygenase 2 (COX-2) activities, and can be deleterious to the host [19,20,21]. COX-2 is a highly inducible enzyme that catalyzes the conversion of arachidonic acid into prostaglandins (PGs) during inflammation. One of the PGs produced at high levels in inflammation is prostaglandin E_2_ (PGE_2_), a major COX-2 product at inflammatory sites, which contributes to local blood flow increases, edema formation, and pain sensitization [22,23].

Five compounds from *Corydalis heterocarpa* were screened for their ability to inhibit LPS-induced NO production in RAW 264.7 macrophages at a concentration of 100 μM. This activity was not attributable to cytotoxicity of **1**–**5** as confirmed by the MTT assay, which showed little cytotoxicity for any of these compounds at the screened concentrations. Among the compounds tested, heterocarpin (**1**) most strongly inhibited NO production, so heterocarpin was further investigated for its inhibitory effect on production of PGE_2_, the major metabolite of the COX-2 pathway, which plays a critical role in the pathogenesis of acute and chronic inflammatory diseases [24,25]. PGE_2_ production was prominently decreased by heterocarpin, showing inhibition rate of 85.5% at a concentration of 100 μM. To confirm the underlying mechanism of this potent effect, we examined its effect on protein expression of both iNOS and COX-2 enzymes, which are known to be responsible for NO and PGE_2_ production, respectively. We found that heterocarpin suppressed LPS-induced iNOS and COX-2 protein expression. This means that the inhibition of NO and PGE_2_ production by heterocarpin is a result of the inhibition of the iNOS and COX-2 expression.

NO and PGE_2_ are known to act as secondary mediators of pro-inflammatory cytokines, such as TNF-α, IL-1β and IL-6, which are considered to be important initiators of the inflammatory response and mediators of the development of various inflammatory diseases [26,27]. Among these, TNF-α is implicated as a key cytokine playing an important role in the immune response such as autoimmune reactions, and its production is crucially required for the synergistic induction of NO synthesis in LPS-stimulated macrophages [28]. IL-1β is also an important mediator of the inflammatory response produced by activated macrophages, and is involved in a variety of cellular activities such as cell proliferation, differentiation, and apoptosis. Likewise, IL-6 plays a role as a crucial pro-inflammatory cytokine, regarded as an endogenous mediator of LPS-induced fever [15,16,29,30,31]. In this study, heterocarpin inhibited production of TNF-α, IL-1β, and IL-6 in a dose dependent manner in LPS-stimulated RAW 264.7 cells. In particular, IL-1β production was greatly decreased by heterocarpin, with an inhibition ratio of 95.7% at a concentration of 100 μM.

In conclusion, these results suggest that heterocarpin suppresses the production of NO, PGE_2_, and pro-inflammatory cytokines TNF-α, IL-1β and IL-6 by reducing their protein expression levels as measured by western blot analysis. Therefore, heterocarpin may be useful for the treatment of inflammatory diseases, although further studies are needed to elucidate the mechanism of its *in vivo* anti-inflammatory activity.

## 3. Experimental Section

### 3.1. General Procedures

Optical rotation was determined on a Perkin-Elmer polarimeter 341 (The Perkin-Elmer Co., Norwalk, CT, USA) using a 1 cm cell. NMR spectra were recorded in CD_3_OD and CDCl_3_ on a Varian Mercury 300 instrument (Varian, Palo Alto, CA, USA) at 300 MHz for ^1^H and 75 MHz for ^13^C using standard pulse sequence programs. All chemical shifts were recorded with respect to TMS as an internal standard. Mass spectroscopic data were obtained at the Korean Basic Science Institute, Seoul, Korea. High performance liquid chromatography (HPLC) was performed with a Dionex P580 (Dionex Corp., Sunnyvale, CA, USA) with a Varian 350 RI detector (Varian, Walnut Creek, CA, USA). All solvents used were spectroscopic grade or were distilled from glass prior to use.

### 3.2. Reagents

Dulbecco’s modified Eagle’s medium (DMEM), fetal bovine serum (FBS), penicillin-streptomycin and other reagents for cell culture were obtained from Gibco (Invitrogen, Carlsbad, CA, USA). 3-(4,5-Dimethylthiazol-2-yl)-5-(3-carboxymethoxphenyl)-2-(4-sulfopheny)-2*H*-tetrazolium (MTS) was obtained from Promega (Madison, WI, USA). Lipopolysaccharide (LPS, from *Escherichia coli*), Griess reagent and phosphate buffer saline (PBS) were purchased from Sigma Chemical Co. (St. Louis, MO, USA). A PGE_2_ immunoassay kit was purchased from Cayman Chemical (Ann Arbor, MI, USA). Mouse TNF-α, IL-1β and IL-6 and ELISA kits were purchased from R&D systems (Minneapolis, MN, USA). PRO-PREP™ Protein Extraction Solution was from iNtRON Biotechnology (Kyungki-Do, Korea). The primary antibody for iNOS was obtained from BD Bioscience (San Jose, CA, USA) and COX-2 was purchased from Cayman Chemical. GAPDH and second antibodies were purchased from Santa Cruz Biotechnology (Santa Cruz, CA, USA). Other chemicals were from Sigma–Aldrich (St. Louis, MO, USA).

### 3.3. Plant Material

Whole plants of *Corydalis heterocarpa* were collected in Muan-gun, Jeollanamdo, Korea in July, 2003. The plant was identified by Sung Gi Moon by its morphological characteristics. A voucher specimen is deposited at the Herbarium of the Department of Marine Environment and Bioscience, Korea Maritime University, Busan, Korea (voucher No. 03H-7).

### 3.4. Extraction and Isolation

The collected samples (300 g) were air-dried, chopped into small pieces, and extracted for 2 days with MeOH (3 L × 2) and CH_2_Cl_2_ (3 L × 2), respectively. The combined crude extracts (41.1 g) were evaporated under reduced pressure and partitioned between CH_2_Cl_2_ and water. The organic layer was further partitioned between 85% aq. MeOH and *n*-hexane, and the aqueous layer was fractionated with *n*-BuOH and H_2_O, successively, to afford *n*-hexane (7.3 g), 85% aqueous MeOH (12.0 g), *n*-BuOH (4.3 g), and water (20.0 g).

A portion of the 85% aqueous MeOH (6.0 g) fraction was subjected to C_18_ reversed-phase vacuum flash chromatography using stepwise gradient mixtures of MeOH and water (50%, 60%, 70%, 80%, 90% aq. MeOH, and 100% MeOH) as eluents to give six subfractions. Fraction 3 was subjected by reversed-phase HPLC (YMC ODS-A, 65% aq. MeOH, 1 cm × 25 cm, S-5 μm, 2 mL/min) to afford three mixtures containing **1** and **4**. Each mixture was further separated by HPLC (YMC ODS-A, 50% aq. AcCN) to give **4** (13.6 mg) and **1** (6.4 mg), respectively.

A half of the *n*-BuOH (2.2 g) fraction was also subjected to C_18_ reversed-phase vacuum flash chromatography using the same solvent mixtures as that of the 85% aqueous MeOH fraction to give six subfractions. Fraction 1 was further separated by Si gel column chromatography with stepwise gradient mixtures of MeOH and CHCl_3_ (0%, 5%, 10%, 20%, 30%, 40%, 50%, 70%, 100% MeOH in CHCl_3_) as eluents to give 9 subfractions. The fourth fraction was separated by preparative TLC on a Si gel chromatograph with EtOAc/MeOH/H_2_O (23:4:3) to afford **5** (6.9 mg) and two mixtures, which were separated by reversed-phase HPLC (YMC ODS-A, 43 and 60% aq. MeOH) to give **2** (6.8 mg) and **3** (4.5 mg), respectively.

*Heterocarpin* (**1**): Amorphous white solid; mp 155–158 °C; ${\text{[α]}}_{\text{D}}^{20}$ +20° (*c* 0.25, MeOH); HRFABMS *m*/*z* 259.0970 [M + H]^+^ (calcd. for C_15_H_14_O_4_, 259.0970); ^1^H-NMR (CD_3_OD) δ: 6.33 (1H, s, H-8), 6.03 (1H, s, H-3), 4.74 (1H, s, H-4′a), 4.68 (1H, s, H-4′b), 4.38 (1H, t, *J* = 6.6 Hz, H-2′), 2.98 (1H, dd, *J* = 6.6, 13.48 Hz, H-1′a), 2.85 (1H, dd, *J* = 6.6, 13.48 Hz, H-1′b), 2.34 (3H, s, 2-Me), 1.81 (3H, s, H-5′); ^13^C-NMR (CD_3_OD) δ: 183.8 (C, C-4), 164.2 (C, C-7), 168.8 (C, C-2), 160.5 (C, C-5), 157.9 (C, C-9), 148.5 (C, C-3′), 110.2 (C, C-6), 111.0 (CH_2_, C-4′), 108.7 (CH, C-3), 104.7 (C, C-10), 94.4 (CH, C-8), 76.2 (CH, C-2′), 29.7 (CH_2_, C-1′), 20.3 (CH_3_, 2-Me), 17.8 (CH_3_, C-5′).

*Cnidimol A* (**2**): Amorphous white solid; ${\text{[α]}}_{\text{D}}^{20}$ −18.00° (*c* 0.50, MeOH); ESIMS *m*/*z* 277 [M + H]^+^; ^1^H-NMR (CD_3_OD) δ: 7.21 (1H, s, H-8), 6.12 (1H, s, H-3), 5.35 (1H, t, *J* = 7.7 Hz, H-2′), 4.31 (2H, s, H-5′), 3.46 (2H, d, *J* = 7.7 Hz, H-1′), 2.31 (3H, s, 2-Me), 1.74 (3H, s, H-4′); ^13^C-NMR (CD_3_OD) δ: 184.3 (C, C-4), 169.9 (C, C-2), 159.3 (C, C-7), 157.6 (C, C-5), 156.6 (C, C-9), 135.8 (C, C-3′), 125.4 (CH, C-2′), 116.6 (C, C-6), 109.1 (CH, C-3), 107.6 (C, C-10), 99.8 (CH, C-8), 61.6 (CH_2_, C-5′), 22.5 (CH_2_, C-1′), 21.5 (CH_3_, C-4′), 20.4 (CH_3_, 2-Me).

*Cnidimoside A* (**3**): Amorphous white solid; ${\text{[α]}}_{\text{D}}^{20}$ −3.16° (*c* 0.32, MeOH); FABMS *m*/*z* 439 [M + H]^+^; ^1^H-NMR (CD_3_OD) δ: 6.29 (1H, s, H-8), 5.98 (1H, s, H-3), 5.46 (1H, t, *J* = 7.0 Hz, H-2′), 4.65 (1H, d, *J* = 12.1 Hz, H-5′a), 4.32 (1H, d, *J* = 12.1 Hz, H-5′b), 4.31 (1H, br.s, H-1′′), 3.89 (1H, dd, *J* = 1.9, 11.6 Hz, H-6′′a), 3.72 (1H, dd, *J* = 4.5, 11.6 Hz, H-6′′b), 3.37 (1H, m, H-4′′), 3.35 (2H, d, *J* = 7.0 Hz, H-1′), 3.34 (1H, m, H-5′′), 3.32 (1H, m, H-3′′), 3.21 (1H, m, H-2′′), 2.31 (3H, s, 2-Me), 1.76 (3H, s, H-4′); ^13^C-NMR (CD_3_OD) δ: 183.6 (C, C-4), 168.6 (C, C-2), 164.0 (C, C-7), 159.6 (C, C-5), 157.6 (C, C-9), 132.3 (C, C-3′), 128.7 (CH, C-2′), 112.1 (C, C-6), 108.6 (CH, C-3), 104.5 (C, C-10), 102.3 (CH, C-1′′), 94.3 (CH, C-8), 78.6 (CH, C-5′′), 77.7 (CH, C-3′′), 75.0 (CH, C-2′′), 71.6 (CH, C-4′′), 68.1 (CH_2_, C-5′), 62.7 (CH_2_, C-6′′), 22.0 (CH_2_, C-1′), 21.8 (CH_3_, C-4′), 20.3 (CH_3_, 2-Me).

*Isopimpinellin* (**4**): Amorphous white solid; ${\text{[α]}}_{\text{D}}^{25}$: +9.52° (*c* 0.21, MeOH); ^1^H-NMR (CD_3_OD) δ: 8.11 (1H, d, *J* = 9.9 Hz, H-4), 7.62 (1H, d, *J* = 2.3 Hz, H-2′), 7.00 (1H, d, *J* = 2.3 Hz, H-1′), 6.28 (1H, d, *J* = 9.9 Hz, H-3), 4.26 (3H, s, H-5-OCH_3_), 4.15 (3H, s, H-8-OCH_3_); ^13^C-NMR (CDCl_3_) δ: 160.4 (C-2), 149.8 (C-7), 145.0 (C-2′), 144.1 (C-5), 143.5 (C-9), 139.3 (C-4), 128.0 (C-8), 114.7 (C-6), 112.7 (C-3), 107.5 (C-10), 105.0 (C-1′), 61.7 (C-8-OCH_3_), 60.8 (C-5-OCH_3_); EIMS *m*/*z* 246 [M]^+^.

*Hyunganol* *II* (**5**): Amorphous white solid; ${\text{[α]}}_{\text{D}}^{20}$ +22.33° (*c* 0.36, MeOH); ^1^H-NMR (CD_3_OD) δ: 6.76 (1H, d, *J* = 8.1 Hz, H-9), 6.17 (1H, d, *J* = 8.1 Hz, H-8), 4.53(1H, t, *J* = 8.9 Hz, H-2′), 3.03 (2H, dd, *J* = 2.3, 8.9 Hz, H-1′), 2.74 (2H, t, *J* = 6.5 Hz, H-4), 2.44 (2H, t, *J* = 6.5 Hz, H-3), 1.20 (3H, s, H-4′), 1.22 (3H, s, H-5′); ^13^C-NMR (CD_3_OD) δ: 182.5 (C-2), 160.8 (C-7), 152.8 (C-5), 130.5 (C-9), 123.1 (C-10), 114.9 (C-6), 101.7 (C-8), 90.7 (C-2′), 72.6 (C-3′), 40.4 (C-3), 29.5 (C-1′), 27.5 (C-4), 25.3 (C-4′), 25.1 (C-5′) ); FABMS *m*/*z* 267 [M + H]^+^.

### 3.5. Cell Culture

A RAW 264.7 murine macrophage cell line was obtained from the Korean Cell Line Bank (Seoul, Korea) and then cultured in DMEM supplemented with 10% FBS, 100 U/mL penicillin, 100 μg/mL streptomycin, and 100 μM MEM non-essential amino acid solution at 37 °C in a humidified atmosphere of 5% CO_2_ and 95% air.

### 3.6. Measurement of Cell Viability

Cell viability was assessed using the CellTiter 96 Aqueous One kit (Promega). Briefly, RAW 264.7 cells were seeded onto a 96 well plate at 5 × 10^4^ cells/well. After incubation with various concentration of compounds, 20 μL of 3-(4,5-dimethylthiazol-2-yl)-5-(3-carboxymethoxyphenyl)-2-(4-sulfopheny)-2*H*-tetrazolium (MTS), which is converted to a formazan product by metabolically active cells, was added to each well. After 2 h of incubation, the optical densities at 490 nm were measured using a microplate reader (Tecan Systems, San Jose, CA, USA). The compounds were considered to be cytotoxic when the optical density of the sample-treated group was less than 90% of that in the control group.

### 3.7. Measurement of Nitrite and PGE2

RAW 264.7 cells (2 × 10^5^ cells/well) were seeded onto a 24-well culture plate at 37 °C for overnight in medium. The cells were preincubated with different concentrations of the compounds for 1 h and then incubated for 24 h with or without LPS. NO production was monitored by measuring nitrite levels in the culture media using Griess reagent (1% sulfanilamide, 0.1% *N*-1-naphthylenediamine dihydrochloride and 2.5% phosphoric acid). The absorbance was measured at 570 nm after incubation for 10 min. The nitrite levels in the samples were calculated from a standard curve generated using known concentrations of sodium nitrite. The PGE_2_ concentration in the culture supernatant was also measured to determine the inhibitory activities of compound 1 using an enzyme immunoassay kit (Cayman Chemical) according to the manufacturer’s instructions.

### 3.8. Enzyme-Linked Immunosorbent Assay (ELISA)

RAW 264.7 cells (2 × 10^5^ cells/well) were plated in a 24-well plate and then incubated with compound 1 for 1 h in the present or absence of LPS (100 ng/mL) for 24 h. The supernatants of cell cultures were used to measure the TNF-α, IL-1β and IL-6 levels using ELISA kits (R&D Systems) according to the manufacturer’s instructions. The cytokine concentration in the samples was calculated from a standard curve developed using a known concentration of recombinant TNF-α, IL-1β and IL-6.

### 3.9. Western Blot Analysis

After activation with LPS, RAW 264.7 cells (2 × 10^6^ cells/well) were washed once with 10 mM PBS (pH 7.4) containing 150 mM NaCl and then lysed with PRO-PREP™ Protein extraction solution (iNtRON Biotechnology). The protein concentration was determined using the Pierce^®^ 660 nm protein assay reagent (Rockford, IL, USA) according to the manufacturer’s instructions. Lysates (20 μg/lane) were separated by 10% SDS-PAGE on polyacrylamide gels and then transferred to polyvinylidene difluoride membranes. Next, the membranes were blocked with 5% non-fat dry milk or BSA in PBS-T solution for 1 h and then probed with iNOS (BD Biosciences) and COX-2 antibodies (Cayman Chemical) overnight at 4 °C. The membranes were washed with PBS-T three times and incubated with a secondary HRP-conjugated antibody for 2 h at room temperature. Following three further washings in PBS-T, the protein bands were visualized using an ECL detection reagent (Pierce).

### 3.10. Statistical Analysis

The data were presented as mean ± SD. The statistical significance of the difference between mean values was determined by the pair *t*-test. *p*-values less than 0.05 or 0.01 were considered statistically significant.
